# The changing epidemiology of Staphylococcus aureus?

**DOI:** 10.3201/eid0702.010204

**Published:** 2001

**Authors:** H F Chambers

**Affiliations:** University of California San Francisco and San Francisco General Hospital, San Francisco, California, USA.

## Abstract

Strains of methicillin-resistant Staphylococcus aureus (MRSA), which had been largely confined to hospitals and long-term care facilities, are emerging in the community. The changing epidemiology of MRSA bears striking similarity to the emergence of penicillinase-mediated resistance in S. aureus decades ago. Even though the origin (hospital or the community) of the emerging MRSA strains is not known, the prevalence of these strains in the community seems likely to increase substantially.


### The Changing Epidemiology of *Staphylococcus aureus*?

**Henry F. Chambers**
University of California San Francisco and San Francisco General Hospital,
San Francisco, California, USA

Strains of methicillin-resistant *Staphylococcus aureus* (MRSA), which had been largely confined to hospitals and long-term care facilities, are emerging in the community. The changing epidemiology of MRSA bears striking similarity to the emergence of penicillinase-mediated resistance in *S. aureus* decades ago. Even though the origin (hospital or the community) of the emerging MRSA strains is not known, the prevalence of these strains in the community seems likely to increase substantially.

Recent reports of strains of methicillin-resistant *Staphylococcus aureus* (MRSA) isolated from children in the community have led to speculation that the epidemiology of *S. aureus* is changing (1-3). Epidemiologic features of the cases described in these reports show a major departure from features typically associated with MRSA colonization or infection. Traditionally, MRSA infections have been acquired almost exclusively in hospitals, long-term care facilities, or similar institutional settings (4). Risk factors for MRSA colonization or infection in the hospital include prior antibiotic exposure, admission to an intensive care unit, surgery, and exposure to an MRSA-colonized patient (4,5).

Humans are a natural reservoir for *S. aureus*, and asymptomatic colonization is far more common than infection. Colonization of the nasopharynx, perineum, or skin, particularly if the cutaneous barrier has been disrupted or damaged, may occur shortly after birth and may recur anytime thereafter (6). Family members of a colonized infant may also become colonized. Transmission occurs by direct contact to a colonized carrier. Carriage rates are 25% to 50%; higher rates than in the general population are observed in injection drug users, persons with insulin-dependent diabetes, patients with dermatologic conditions, patients with long-term indwelling intravascular catheters, and health-care workers (7). Young children tend to have higher colonization rates, probably because of their frequent contact with respiratory secretions (8,9). Colonization may be transient or persistent and can last for years (10).

When cases of MRSA infection have been identified in the community, a thorough investigation usually reveals a history of recent hospitalization; close contact with a person who has been hospitalized; or other risk factors, such as previous antimicrobial-drug therapy (11,12). In the 1980-1981 outbreak of community-acquired MRSA infections in Detroit (13,14), approximately two thirds of the patients affected were injection drug users. Previous antimicrobial therapy was associated with infection by a strain of MRSA. Recent hospitalization, defined as within 4 months (which may not have been long enough, given that hospital-acquired MRSA colonization may last years [10]), was not a predictor of MRSA infection in the drug users; however, the epidemic strain had the same phage type as a strain of MRSA responsible for an outbreak in a burn unit in Minnesota in 1976 (15). The source of the Detroit outbreak was not identified. Frequent needle sharing was speculated to be the mode of transmission in the community. In contrast to infection in injection drug users, MRSA infection in nonusers was strongly associated with recent hospitalization, which suggests that drug users had become colonized during a previous hospital admission. In turn, patients (and probably health-care workers, who become colonized with MRSA as a consequence of their exposure to colonized patients) in a hospital or other health-care setting can then transmit MRSA strains to close associates and family members by direct contact.

Direct or indirect exposure to an institutional health-care setting in which MRSA is likely to be found and other risk factors typically associated with MRSA colonization are strikingly absent from the recently described cases in which MRSA seems to have been acquired from a community reservoir. The antimicrobial susceptibility patterns observed for these MRSA strains are further evidence of a possible community origin. Unlike hospital strains, which typically are resistant to multiple antibiotics and can be shown by typing schemes to be related to other hospital strains, these so-called community strains have tended to be susceptible to other antibiotic classes and often are resistant only to beta-lactam antibiotics (1,2,9). The lack or loss of resistance to multiple antibiotics suggests a community origin because antibiotic selective pressure is much lower within the community than in hospitals, and the survival advantage of multiple-drug resistance is lower. Typing by pulsed-field gel electrophoresis (PFGE) also suggests that these strains are distinctive.

### Emergence of Penicillinase-Producing *S. aureus*

Whether their appearance in the community and their susceptibility to antibiotics other than beta-lactams are fundamental changes in MRSA epidemiology is debatable. The epidemiology of MRSA and the factors driving resistance bear strong similarities and parallels to those occurring with

Address for correspondence: Henry F. Chambers, Infectious Diseases Laboratories, Fourth Floor, Building 30, San Francisco General Hospital, 1001 Potrero Avenue, San Francisco, CA 94110, USA; fax: 415-648-8425; e-mail: chipc@itsa.ucsf.edu penicillin-resistant strains of *S. aureus* in the 1940s and 1950s. When Kirby's first description of penicillinase-producing strains of *S. aureus* was published in 1944 (16), resistance was infrequently encountered, with only a handful of strains available for study. As with MRSA, penicillinase-producing strains first were isolated from hospitalized patients (17). Community strains tended to be penicillin susceptible. The prevalence of penicillinase-producing strains of *S. aureus* within hospitals soon began to rise as penicillin became readily available after World War II. Within a few years, most hospital isolates were resistant to penicillin (17). As was observed decades later with MRSA, previous treatment with a beta-lactam antibiotic, in this case penicillin, increased the chances of isolating a penicillin-resistant strain. Colonization of hospital staff by penicillin-resistant strains and their role in transmission also were notable features of these early reports.

Although penicillinase-producing strains were universally present in hospitals by the early 1950s, community isolates of *S. aureus* were considered to be largely penicillin susceptible. Penicillin continued to be recommended as an effective anti-staphylococcal agent as late as the early 1970s (18). However, then as now, there was no systematic surveillance for antibiotic resistance among *S. aureus* isolates circulating within communities. The first comprehensive description and accurate assessment of the epidemiology of drug-resistant strains of *S. aureus* were published in 1969 by Jessen et al. (19). Examination of more than 2,000 blood culture isolates of *S. aureus* received at the Statens Seruminstitut in Copenhagen for 1957 to 1966 for which detailed information on the origin of infection (hospital or community) was available confirmed a high prevalence of penicillin resistance (85% to 90%) for hospital isolates of *S. aureus*. Somewhat unexpected was that penicillinase-producing strains were almost as common in the community, with 65% to 70% of isolates resistant to penicillin. The community-acquired isolates often were resistant only to penicillin, whereas nosocomial strains typically were resistant to multiple antibiotics.

By the 1970s, it was apparent that the high prevalence of penicillin resistance among community isolates was not limited to Denmark. A remarkably constant 70% to 85% prevalence of penicillinase-producing strains was found regardless of location in inner cities, suburbs, rural areas, within and outside the United States (8,20,21). A population-based study conducted in 1972 revealed that 47% of healthy school-aged children under 10 years of age were carriers of *S. aureus* and that 68% of colonizing strains were penicillin-resistant (8).

Staphylococcal resistance was reported shortly after penicillin was introduced, and within approximately 6 years, 25% of hospital strains were resistant (Table 1). One to two decades later, 25% of community isolates were penicillin resistant (22, 23). Although the rates are only approximate because they are based on reports from numerous locations, a clear correlation exists between the prevalence of penicillin-resistant strains of *S. aureus* reported in hospitals and rates in the community (Figure). The upswing in community rates followed soon after nosocomial rates exceeded 40% to 50%, and by the 1970s, the two rates were practically equal.

Table 1. Time required for prevalence rates of resistance to reach 25% in hospitals

| Drug | Year drug introduced | Years to report of resistance | Years until 25% rate in hospitals | Years until 25% rate in community |
|---|---|---|---|---|
| Penicillin | 1941 | 1-2 | 6 | 15-20 |
| Vancomycin | 1956 | 40 | ? | ? |
| Methicillin | 1961 | <1 | 25-30 | 40-50 (projected) |

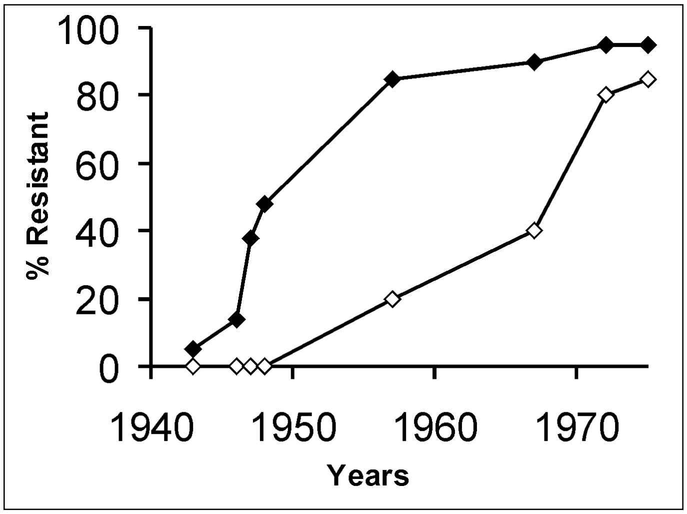

Figure. Secular trends of approximate prevalence rates for penicillinase-producing, methicillin-susceptible strains of *Staphylococcus aureus* in hospitals (closed symbols) and the community (open symbols).

### Community-Acquired MRSA

In the past two decades, the prevalence of MRSA strains has steadily increased in hospitals in the United States and abroad. National Nosocomial Infections Surveillance (NNIS) data collected by the Centers for Disease Control in the early to mid-1980s indicated that MRSA was limited mainly to relatively large urban medical centers and that rates were 5% to 10%. Smaller, nonreferral centers were relatively free of MRSA, with prevalence rates well below 5%. By the 1990s, rates among these smaller (<200-bed) community hospitals had increased to 20%, and twice that rate was found in the larger urban centers. More recent surveillance data from NNIS indicate that rates have continued to rise, with the prevalence of MRSA isolates from intensive care units approaching 50% by the end of 1998. Unless this upward trend has reversed, the prevalence rate of MRSA in U.S. hospitals likely has reached 50%. At these high rates, the emergence of correspondingly high rates of MRSA strains in the community can be anticipated. Because no systematic, population-based surveillance of community isolates of *S. aureus* exists, the true prevalence of MRSA cannot be determined. One hospital-based study found that up to 40% of MRSA infections in adults were acquired before admission to the hospital (24). Published reports of MRSA colonization and infection among study participants who lack traditional risk factors indicate that community prevalence rates are rising. For the period 1976 through 1990, a Medline search identified 10 articles in which key words "methicillin-resistant *Staphylococcus aureus*" and "community" appeared in the title (Table 2). For the period 1991 through 1999, 39 articles were identified; 29 were published from 1996 through 1999. A community-based survey of injection drug users in the San Francisco Bay area communities found that up to 35% of *S. aureus* carriers harbored MRSA (Table 3).

In early reports, community isolates of MRSA had affected persons with known risk factors for colonization (contact with health-care facilities, previous antimicrobial therapy), whereas more recent reports describe colonization and transmission in populations lacking risk factors. A recent study of methicillin-resistant *S. aureus* carriage in children attending day-care centers is reminiscent of Ross's survey of healthy children colonized with penicillin-resistant *S. aureus* strains two decades earlier (9). This survey of two day-care centers in Dallas, Texas, each of which had an index case of MRSA infection, revealed that 3% and 24% of children in the respective centers were colonized. The isolates generally were susceptible to multiple antibiotics, which is in contrast to the typical, multiple-drug-resistant hospital isolate. Forty percent of the children colonized had had no contact with a health-care facility or a household member with such contact within the previous 2 years, which suggests that sustained transmission and colonization of MRSA in children were occurring in the community. A study from Chicago found a 25-fold increase in the number of children admitted to the hospital with an MRSA infection who lacked an identifiable risk factor for prior colonization (1). These MRSA strains, also presumably transmitted and acquired in a community setting, tended to be susceptible to multiple antibiotics. Two examined strains had PFGE patterns that were distinct from the common nosocomial isolates.

Table 2. Estimated prevalence of methicillin-resistant *Staphylococcus aureus* strains in U.S. hospitals and publications[a] pertaining to community-acquired methicillin-resistant *S. aureus*

| Years | Hospital prevalence rate (%) | Total no. of of articles | No. of articles pertaining to children | No. of articles pertaining to other groups |
|---|---|---|---|---|
| 1996-1999 | 40 | 29 | 8 | 3 (seniors, rugby team, wrestlers) |
| 1991-1995 | 28 | 10 | 0 | 0 |
| 1986-1990 | 20 | 5 | 1 | 0 |
| 1981-1985 | 5 | 5 | 0 | 4 (addicts) |
| 1976-1980 | <5 | 0 | 0 | 0 |

[a]Identified by Medline search.

Table 3. Outpatient population-based prevalence of *Staphylococcus aureus* carriage and percentage of carriers with methicillin-resistant (MRSA) strains among injection drug users

| Location | *S. aureus* carriage (%) | Carriers with MRSA (%) |
|---|---|---|
| San Francisco | | |
| Western addition | 25 | 16 |
| Tenderloin | 20 | 21 |
| Mission | 34 | 35 |
| Bayview | 23 | 12 |
| East Bay | | |
| Oakland | 18 | 12 |
| Richmond | 20 | 6 |

The deaths of four children from rural Minnesota and North Dakota caused by infection with community-acquired MRSA strains brought the problem to national attention in 1999 (2). These children, like those in the Chicago study, lacked risk factors for MRSA infection. The infections were caused by strains susceptible to several antibiotics, except beta-lactams. The PFGE patterns of these strains indicated that they were related to one another but differed from typical nosocomial isolates circulating in local hospitals.

These reports of infection and colonization by strains of MRSA in children provide compelling evidence that MRSA strains, like penicillinase-producing strains almost 30 years ago, have gained a foothold in the community and are emerging as important outpatient pathogens. Based on the experience with penicillin-resistant strains, prevalence of MRSA among community isolates may be as high as 25% within the next 5 to 10 years (Table 1).

### Origins of Community-Acquired MRSA

The origins of these community-acquired strains are subject to debate. One possibility is that they are feral descendants of hospital isolates. If so, these isolates must have undergone considerable change because they possess distinctive PFGE patterns and have lost resistance to multiple antibiotics. Another possibility is that the community isolates arose as a consequence of horizontal transfer of the methicillin-resistance determinant into a formerly susceptible background. This possibility could also account for the unique PFGE patterns and lack of resistance to multiple drugs. In the case of penicillinase-mediated resistance, dissemination of strains from the hospital and horizontal transfer of the penicillinase gene into susceptible recipient strains were both likely to have contributed to emergence of penicillin-resistant strains in the community. Penicillinase typically is plasmid encoded and can be readily transferred by transduction or conjugation. These characteristics account for methicillin-susceptible, penicillinase-producing strains being genetically diverse and polyclonal.

Unlike plasmid-encoded penicillinase, the methicillin resistance determinant, *mec*, is chromosomally encoded. Horizontal transfer of *mec* is thought to be relatively rare; only a handful of ancestral strains account for all clinical isolates worldwide (25). Ribotyping (a genotyping scheme that uses Southern blot analysis to identify DNA restriction enzyme polymorphisms of the five to six ribosomal RNA genes distributed throughout the *S. aureus* chromosome) and cluster analysis indicate that *mec* has integrated into at least three distinct methicillin-susceptible chromosomal backgrounds, A, B, and C (26, 27). *mec* itself is polymorphic; three types have been identified: I, II, and III. These polymorphs differ in number of base pairs, genetic organization, number of insertion sequences, and resistance determinants (Table 4). All three mec types have been found integrated into ribotype cluster A. Type II *mec* has also integrated into cluster B and C ribotype backgrounds. Thus, five distinct clones of MRSA have been identified worldwide since the first strain was isolated in the United Kingdom in 1961; even if more clones were identified, the relatively low number pales in comparison to the large number of distinct clones of methicillin-susceptible clones.

Table 4. Elements found within three types of *mec*-associated DNA

| | *mec* types | | |
|---|---|---|---|
| Genetic feature[a] | I | II | III |
| Size | 32 kb | 52 kb | 60 kb |
| *mecA* | + | + | + |
| *mecR1-mecI* | - | + | + |
| *ccrAB* | + | + | + |
| pUB110 | - | + | - |
| IS431 (number) | 1 | 2 | 4 |
| Tn554 (number) | 0 | 1 | 2 |
| Tc, Hg resistance | - | - | + |

[a]mecA = gene encoding PBP 2a, the penicillin-binding protein with low binding affinity that mediates methicillin resistance; *mecR1-mecI* = sensor-transducer and repressor genes that regulate production of inducible PBP 2a; *ccrAB* = cassette chromosome recombinases A and B that mobilize the *mec* element; pUB110 = integrated plasmid that encodes tobramycin and kanamycin resistance; IS431 = insertion sequence; Tn554 = erythromycin-resistance encoding transposon; Tc = tetracycline-resistance determinant; Hg = mercury-resistance determinant.

Unlike the mechanisms responsible for horizontal transfer of penicillinase resistance, the mechanism by which *mec* might be mobilized and transferred had not been understood until recently. Hiramatsu and co-workers have identified two genes, *ccrAB* (cassette chromosome recombinase genes A and B), which are homologous to DNA recombinases of the invertase-resolvase family and can mobilize *mec* (28). The proteins encoded by these genes catalyze precise excision and precise site-specific and orientation-specific integration of *mec* into the *S. aureus* chromosome. Thus, *mec* is somewhat analogous to the pathogenicity islands found in gram-negative bacilli, except that this locus encodes resistance determinants instead of virulence factors. How an element as large as *mec* is transferred from donor to recipient is not known. Nevertheless, as the prevalence of MRSA strains has increased, so has the abundance of *mec* DNA. Even though transfer of *mec* occurs rarely, the chances that it might occur have correspondingly increased. The community-acquired strains could possibly have arisen as a consequence of one of these rare transfers of *mec* from a nosocomial donor into a susceptible recipient. With appropriate analysis of *mec* DNA and the recipient chromosome, researchers should be able to determine whether these newly identified community-acquired strains are feral or freestanding. Regardless of the origins, which are likely to become obscured as clones move back and forth between hospital and community over time, emergence of MRSA within the community is a major threat with several important clinical implications: treatment failure with accompanying complications or death may result if an antistaphylococcal beta-lactam antibiotic is used and the infecting strain proves to be resistant; infections caused by methicillin-resistant strains may be more difficult to manage or more expensive to treat, perhaps because vancomycin is inherently less efficacious (29-33); and the increasing prevalence of MRSA will inevitably increase vancomycin use, adding further to the problem of antibiotic-resistant gram-positive bacteria.

Antimicrobial resistance to penicillin, methicillin, or vancomycin is an unavoidable consequence of the selective pressure of antibiotic exposure. Although the details of the epidemiology of staphylococcal drug resistance may change, the fundamental forces driving it are similar. The question is not whether resistance will occur, but how prevalent resistance will become. Minimizing the antibiotic pressure that favors the selection of resistant strains is essential to controlling the emergence of these strains in the hospital and the community, regardless of their origins.

This work was supported by United States Public Health Service grant AI46610 from NIH/NIAID.

Dr. Chambers is professor of medicine at the University of California, San Francisco, and Chief of Infectious Diseases at San Francisco General Hospital. His research interests are staphylococcal infections, experimental therapeutics, and bacterial resistance to antimicrobial agents, particularly to beta-lactam antibiotics.

### References

1. Herold BC, Immergluck LC, Maranan MC, Lauderdale DS, Gaskin RE, Boyle-Vavra S, et al. Community-acquired methicillin-resistant *Staphylococcus aureus* in children with no identified predisposing risk [see comments]. JAMA 1998;279:593-8.
2. CDC. Four pediatric deaths from community-acquired methicillin-resistant *Staphylococcus aureus*--Minnesota and North Dakota, 1997-1999. MMWR Morb Mortal Wkly Rep 1999;48:707-10.
3. Boyce JM. Are the epidemiology and microbiology of methicillin-resistant *Staphylococcus aureus* changing? [editorial; comment]. JAMA 1998;279:623-4.
4. Thompson RL, Cabezudo I, Wenzel RP. Epidemiology of nosocomial infections caused by methicillin-resistant *Staphylococcus aureus*. Ann Intern Med 1982;97:309-17.
5. Boyce JM. Methicillin-resistant *Staphylococcus aureus*: detection, epidemiology, and control measures. Infect Dis Clin North Am 1989;3:901-13.
6. Payne MC, Wood HF, Karakawa W, Gluck L. A prospective study of staphylococcal colonization and infections in newborns and their families. Am J Epidemiol 1966;82:305-16.
7. Wadlvogel FA. *Staphylococcus aureus* (including staphylococcal toxic shock). In: Mandell GL, Bennett JE, Dolin R, editors. Principles and practice of infectious diseases. 5th ed. Philadelphia: Churchill Livingstone, 2000. p. 2072-3.
8. Ross S, Rodroguez W, Controni G, Khan W. Staphylococcal susceptibility to penicillin G: The changing pattern among community isolates. JAMA 1974;229:1075-7.
9. Adcock PM, Pastor P, Medley F, Patterson JE, Murphy TV. Methicillin-resistant *Staphylococcus aureus* in two child care centers. J Infect Dis 1998;178:577-80.
10. Sanford MD, Widmer AF, Bale MJ, Jones RN, Wenzel RP. Efficient detection and long-term persistence of the carriage of methicillin-resistant *Staphylococcus aureus*. Clin Infect Dis 1994;19:1123-8.
11. Gross-Schulman S, Dassey D, Mascola L, Anaya C. Community-acquired methicillin-resistant *Staphylococcus aureus* [letter; comment]. JAMA 1998;280:421-2.
12. L'Heriteau F, Lucet JC, Scanvic A, Bouvet E. Community-acquired methicillin-resistant *Staphylococcus aureus* and familial transmission [letter]. JAMA 1999;282:1038-9.
13. Saravolatz LD, Pohlod DJ, Arking LM. Community-acquired methicillin-resistant *Staphylococcus aureus* infections: a new source for nosocomial outbreaks. Ann Intern Med 1982;97:325-9.
14. Saravolatz LD, Markowitz N, Arking L, Pohlod D, Fisher E. Methicillin-resistant *Staphylococcus aureus*. Epidemiologic observations during a community-acquired outbreak. Ann Intern Med 1982;96:11-16.
15. Crossley K, Landesman B, Zaske D. An outbreak of infections caused by strains of *Staphylococcus aureus* resistant to methicillin and aminoglycosides. II. Epidemiologic studies. J Infect Dis 1979;139:280-7.

16. Kirby WMM. Extraction of a highly potent penicillin inactivator from penicillin resistant staphylococci. Science 1944;99:452-3.
17. Barber M, Rozwadowska-Dowzenko M. Infection by penicillin-resistant staphylococci. Lancet 1948;1:641-4.
18. Weinstein L. The penicillins. In: Goodman L, Gilman A, editors. The pharmacologic basis of therapeutics. New York: Macmillan; 1975. p. 1153.
19. Jessen O, Rosendal K, Bulow P, Faber V, Eriksen KR. Changing staphylococci and staphylococcal infections: A ten-year study of bacteria and cases of bacteremia. N Engl J Med 1969;281:627-35.
20. Hughes GB, Chidi CC, Macon WL. Staphylococci in community-acquired infections: Increased resistance to penicillin. Ann Surg 1976;183:355-7.
21. Hahn DL, Baker WA. Penicillin G susceptibility of "rural" *Staphylococcus aureus.* J Fam Pract 1980;11:43-6.
22. Gould JC, Cruikshank JD. Staphylococcal infection in general practice. Lancet 1957;2:1157-61.
23. Harris DM, Wise PJ. Penicillinase producing staphylococci in general practice and their control by cloxacillin. Practitioner 1969;203:207-11.
24. Layton MC, Hierholzer WJ, Jr, Patterson JE. The evolving epidemiology of methicillin-resistant *Staphylococcus aureus* at a university hospital. Infect Control Hosp Epidemiol 1995;16:12-17.
25. Kreiswirth B, Kornblum J, Arbeit RD, Eisner W, Maslow JN, McGeer A, et al. Evidence for a clonal origin of methicillin resistance in *Staphylococcus aureus*. Science 1993;259:227-30.
26. Hiramatsu K. Molecular evolution of MRSA. Microbiol Immunol 1995;39:531-43.
27. Hiramatsu K, Ito T, Hanaki H. Mechanisms of methicillin and vancomycin resistance in *Staphylococus aureus*. Baillieres Clinical Infectious Diseases 1999;5:221-42.
28. Katayama Y, Ito T, Hiramatsu K. A new class of genetic element, staphylococcus cassette chromosome *mec*, encodes methicillin resistance in *Staphylococcus aureus.* Antimicrob Agents Chemother 2000;44:1549-55.
29. Small PM, Chambers HF. Vancomycin for *Staphylococcus aureus* endocarditis in intravenous drug users. Antimicrob Agents Chemother 1990;34:1227-31.
30. Levine DP, Fromm BS, Reddy BR. Slow response to vancomycin or vancomycin plus rifampin in methicillin-resistant *Staphylococcus aureus* endocarditis [see comments]. Ann Intern Med 1991;115:674-80.
31. Soriano A, Martinez JA, Mensa J, Marco F, Almela M, Moreno-Martinez A, et al. Pathogenic significance of methicillin resistance for patients with *Staphylococcus aureus* bacteremia. Clin Infect Dis 2000;30:368-73.
32. Gentry CA, Rodvold KA, Novak RM, Hershow RC, Naderer OJ. Retrospective evaluation of therapies for *Staphylococcus aureus* endocarditis. Pharmacotherapy 1997;17:990-7.
33. Conterno LO, Wey SB, Castelo A. Risk factors for mortality in *Staphylococcus aureus* bacteremia. Infect Control Hosp Epidemiol 1998;19:32-7.
